# The complete mitochondrial genome of the *Papilio paris* (Lepidoptera: Papilionidae)

**DOI:** 10.1080/23802359.2020.1715281

**Published:** 2020-01-20

**Authors:** Yu Sun, Hua Huang, Xiaojuan Zhang, Jun Xia, Jian Geng, Kai Zhang

**Affiliations:** a Department of Health Inspection and Quarantine, School of Laboratory Medicine, Bengbu Medical College, Bengbu, China;; b Research Center of Clinical Laboratory Science, Bengbu Medical College, Bengbu, China;; c Department of Stomatology, The First Affiliated Hospital of Bengbu Medical College, Bengbu Medical College, Bengbu, China

**Keywords:** Mitogenome, phylogenetic analyses, *Papilio paris*

## Abstract

In the present study, the complete sequence of the mitochondrial genome (mitogenome) of *Papilio paris* (Lepidoptera: Papilionidae) is described. The mitogenome (15,347 bp) of *P. paris* encodes 13 protein-coding genes (PCGs), 22 transfer RNA genes (tRNAs), two ribosomal RNA genes (rRNAs), and an adenine (A) + thymine (T)-rich region. Its gene complement and order is similar to that of other sequenced Lepidopterans. Phylogenetic analyses based on 13 PCGs using maximum-likelihood (ML) revealed that *P. paris* resides in the Papilionoidea family. This study provided the valuable evidence on phylogenetic relationship of the *P. paris* at the molecular level and essential resource for further research on this species.

*Papilio paris* (Lepidoptera: Papilionidae) is a butterfly from the Australasia/Indomalaya (Australia) ecozone. This butterfly can be observed from India until China and also from China until Indonesia. *Papilio paris* is mainly distributed in thickets on slopes and broad-leaved forests, such as rue family plants. The species is one of the important leaf defoliators on *Melicope pteleifolia*. It has five generations a year in Guangzhou, South China, and the generations overlap. The 1–3 instar larvae mainly feed on the young leaves. After the 3th instar larvae, both young leaves and old leaves are fed. When the density of the insect population is large, the young shoots also are taken. It affects the growth of the plants. Moreover, *P. paris* is also one of the most ornamental butterflies. To date, there was less information about the complete mitochondrial genomes of *P. paris* in GenBank. In our study, the complete mitogenome of *P. paris* was determined, which would be useful for further genetic studies, phylogenetic analysis, and conservation of this species.

The *P. paris* specimens were collected from Dashushan Forest Park (31°50′53.03″N, 117°11′1.01″E), Anhui Province, China. The collected specimens were identified as *P. paris* based on the morphological characters by the taxonomist of the Department of Entomology, Anhui Agricultural University, Hefei, China (AHAU). The specimen was stored in 100% ethanol at −80 °C in Bengbu Medical College, Bengbu, China with an accession number: P20160502. The total DNA was extracted using the Genomic DNA Extraction Kit (Aidlab Co., Beijing, China) to the manufacturer instructions. The sequence was amplified using PCR with 12 pairs of primer.

In general, the length of this sequence is 15,347 bp (Genbank No: MN629008), containing two ribosomal RNA genes, 22 putative transfer RNA (tRNA) genes, 13 protein-coding genes, and an adenine (A) + thymine (T)-rich region consisted with previous studies (Sun et al. 2016). The 12 PCGs initiated by ATN codons except for cytochrome c oxidase subunit 1 (*cox1*) gene that is seemingly initiated by the CGA codon as documented in other insect mitogenomes (Dai et al. 2015; Sun, Zhang, et al. 2017). The mitogenome of *P. paris* contains 22 tRNAs (ranging from 61 to 71 nucleotides in length) commonly present in most of Lepidopteran mitogenomes. The 16S rRNA (1325 bp) and 12S rRNA (767 bp) were separated by tRNA^Val^ (Wu et al. 2013). The A + T-rich region is 533 bp long and contains some conserved regions, including ‘ATAGA’ motif followed by a 11 bp poly-T stretch and a microsatellite-like element (AT)_8_. The length of poly-T stretch varies from species to species(Dai et al. 2015; Sun 2019), whereas ATAGA region is conserved in Lepidoptera species (Sun, Chen, et al. 2017).

To reconstruct the phylogenetic relationship among Lepidopteran insects, the nucleotide sequences of the 13 PCGs were first aligned and then concatenated; 31 complete mitogenomes were downloaded from the GenBank database. We reconstructed the phylogenetic relationships using Maximum-Likelihood (ML) method based on concatenated nucleotide sequences of the 13 PCGs of the related Lepidopteran superfamilies. The mitogenomes of *Drosophila melanogaster* (U37541.1) (Lewis et al. 1995) and *Locusta migratoria* (NC_001712) (Flook et al. 1995) were used as outgroup. The multiple alignments of the 13 PCGs concatenated nucleotide sequences were conducted using ClustalX version 2.0. (Thompson et al. 1997). Then, a concatenated set of nucleotide sequences from the 13 PCGs was used for phylogenetic analyses, which were performed using the ML method with the MEGA X program. The phylogenetic analysis reveals that Bombycoidea, Noctuoidea, Geometroidea, Pyraloidea, Papilionoidea, Tortricoidea, Yponomeutoidea, and Hepialoidea families are monophyletic, and Pyraloidea is more closely related to the Papilionoidea superfamiliy. The different species of same family forms a single cluster; moreover, *P. paris* was found closely related to *P. syfanius* of the Papilionoidea (Figure 1). The results are consistent with previous studies. It is concluded from the results that further studies are required to confirm the phylogentic relationships among these superfamiles.

**Figure 1.**
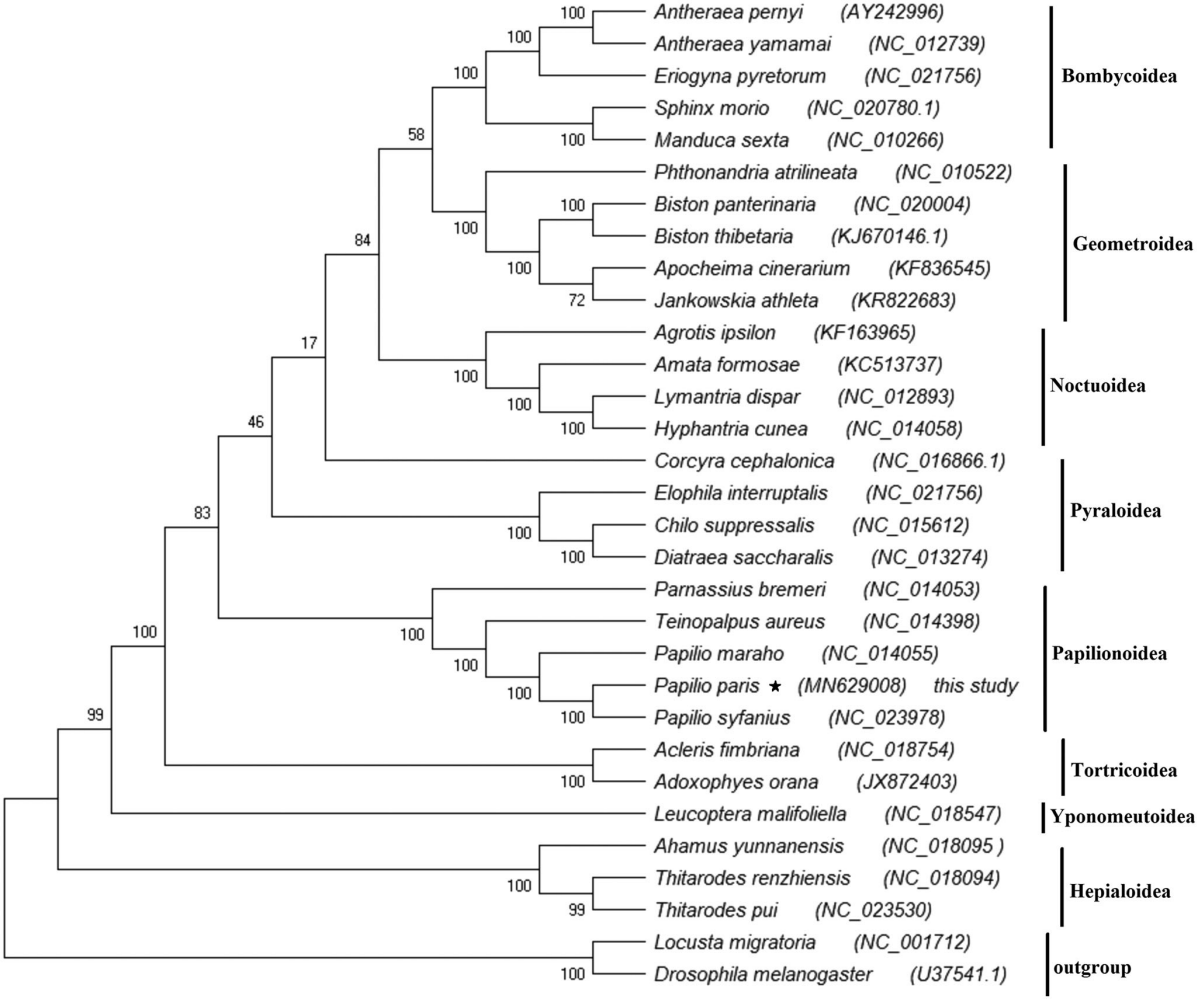
Tree shows the phylogenetic relationships among Lepidopteran insects, constructed using Maximum-Likelihood method. Bootstrap values (1000 repetitions) of the branches are indicated. *Drosophila melanogaster* (U37541.1) and *Locusta migratoria* (NC_001712) were used as outgroups.
